# Comparison of 96-kV and 120-kV cone-beam CT for the assessment of cochlear implants

**DOI:** 10.1186/s12880-024-01322-4

**Published:** 2024-06-13

**Authors:** Iris Burck, Ibrahim Yel, Simon Martin, Moritz H. Albrecht, Vitali Koch, Christian Booz, Daniel Pinto dos Santos, Benjamin Kaltenbach, Hanns Ackermann, Juha Koivisto, Silke Helbig, Timo Stöver, Thomas J. Vogl, Jan-Erik Scholtz

**Affiliations:** 1 Department of Diagnostic and Interventional Radiology, University Hospital Frankfurt, Theodor-Stern-Kai 7, 60590 Frankfurt, Germany; 2 Institute of Biostatistics and Mathematical Modeling, University Hospital Frankfurt, Frankfurt, Germany; 3 Department of Otorhinolaryngology, University Hospital Frankfurt, Frankfurt, Germany; 4 University of Helsinki, Helsinki, Finland

**Keywords:** Cone-Beam Computed Tomography, Cochlear Implant, Radiation Dose

## Abstract

**Background:**

To compare the diagnostic value of 120-kV with conventional 96-kV Cone-Beam CT (CBCT) of the temporal bone after cochlear implant (CI) surgery.

**Methods:**

This retrospective study included CBCT scans after CI surgery between 06/17 and 01/18. CBCT allowed examinations with 96-kV or 120-kV; other parameters were the same. Two radiologists independently evaluated following criteria on 5-point Likert scales: osseous spiral lamina, inner and outer cochlear wall, semi-circular canals, mastoid trabecular structure, overall image quality, metal and motion artefacts, depiction of intracochlear electrode position and visualisation of single electrode contacts. Effective radiation dose was assessed.

**Results:**

Seventy-five patients (females, *n* = 39 [52.0%], mean age, 55.8 ± 16.5 years) were scanned with 96-kV (*n* = 32, 42.7%) and 120-kV (*n* = 43, 57.3%) protocols including CI models from three vendors (vendor A *n* = 7; vendor B *n* = 43; vendor C *n* = 25). Overall image quality, depiction of anatomical structures, and electrode position were rated significantly better in 120-kV images compared to 96-kV (all p < = 0.018). Anatomical structures and electrode position were rated significantly better in 120-kV CBCT for CI models from vendor A and C, while 120-kV did not provide improved image quality in CI models from vendor B. Radiation doses were significantly higher for 120-kV scans compared to 96-kV (0.15 vs. 0.08 mSv, *p* < 0.001).

**Conclusions:**

120-kV and 96-kV CBCT provide good diagnostic images for the postoperative CI evaluation. While 120-kV showed improved depiction of temporal bone and CI electrode position compared to 96-kV in most CI models, the 120-kV protocol should be chosen wisely due to a substantially higher radiation exposure.

## Introduction

Radiological imaging is essential before and after cochlear implant (CI) treatment to guide surgical strategy, ensure quality and recognize complications in order to provide optimal hearing results after CI surgery. The main aim of postoperative imaging is to provide information about correct position of the implanted electrodes and detection or ruling out of insertion trauma. Abnormalities of the intracochlear array course may affect hearing and may result in additional morbidity, hospitalization, and even reoperation [1–5].

In addition to computed tomography (CT) [6, 7], Cone-Beam CT (CBCT) has become an established diagnostic imaging tool to visualize the temporal bone. CBCT provides isotropic resolution with a thinner slice thickness than conventional CT, thus, enhancing detailed imaging of small structures. There is a trend to use postoperative CBCT as a tool for customizing cochlear implants by determining electrode contact locations and deriving patient-specific center frequency mapping, which may potentially optimize place-pitch mismatch and outcomes [8–10]. According to different CBCT manufacturers, CBCT is also supposed to allow a 5-10-fold reduction in radiation exposure compared to conventional CT of the skull [11–14].

Due to lower investment costs and comparatively small space requirement and a more patient-friendly workflow, CBCT has become more popular and widespread than conventional CT. Besides to standard 96-kV protocols, some CBCT scanner models provide protocols with an increased tube voltage of 120-kV. It is expected that image quality using 120-kV is higher with an increase of radiation exposure, when other scan parameters are kept constant. To our knowledge, the additional value of an increased tube voltage of 120-kV in CBCT after CI has not been evaluated so far.

The purpose of this study was to evaluate and compare image quality and diagnostic value of dedicated CBCT datasets obtained with 96-kV and 120-kV after CI surgery.

## Materials and methods

### Study design and patient characteristics

**Fig. 1:**
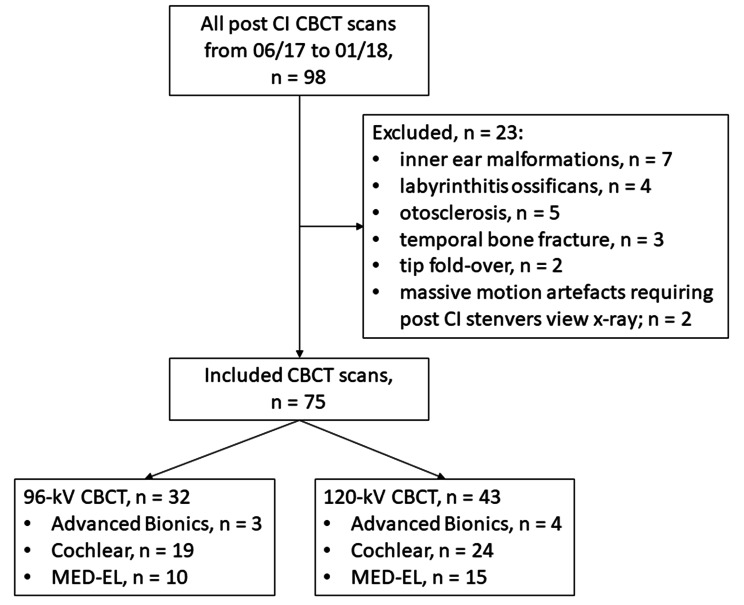
Flow chart. CBCT, cone-beam computed tomography; Advanced Bionics (Sonova Holding AG); Cochlear (Cochlear Limited); Med-El (MED-EL GmbH).

In this retrospective study we included all CBCT exams from June 2017 to January 2018. The data were further analysed after completion of a phantom dose study by the manufacturer. The choice of the most accurate electrode was made individually based on the audiological tests, the etiology of the hearing loss and the anatomy of the cochlea and surgical considerations. We excluded cases with anatomical abnormalities or other cochlear disorders like otosclerosis, labyrinthine fracture, cochlear damage caused by meningitis, calcification of the scala tympani, an intralabyrinthine schwannoma, intracochlear electrode misinsertion, or non-diagnostic images due to extensive motion artefacts (Fig. 1). The included patients were referred for routine postoperative CBCT after CI surgery and randomly assigned to our 96-kV or 120-kV scan protocol. Six different CI electrode-array types from three manufacturers (vendor A: *Advanced Bionics LLC*; vendor B: *Cochlear Ltd*; vendor C: *MED-EL*) were implanted [15–18].

### Scan protocol

All petrous bone examinations were performed with a new-generation CBCT scanner (Planmeca ProMax 3D Max, Planmeca Oy). While tube voltage was set to either 96-kV or 120-kV, all other scan parameters were similar: tube-current, 7.1 mA; effective acquisition time, 10 s; field of view (FOV), 100 × 90 mm with voxel sizes between 100 and 200 cm. For X-ray beam filtration, 2.5 mm aluminum (Al) and 0.5 mm copper (Cu) layers were used. No metal artifact reduction algorithm was used.

For image reconstructions in orthogonal (axial, coronal, and sagittal) views, a dedicated integrated 3D-postprocessing workstation as part of the CBCT device was used. In addition, datasets were transferred to a dedicated workstation (syngo MultiModality Workplace, Siemens) to generate additional oblique multiplanar reformations in Stenvers view for the evaluation of the intracochlear position of the CI electrodes on a cochlear coordinate system showing the complete basal turn of the cochlea in a cross-section plane, as suggested in the recommendations of the international consensus panel [19]. For a more accurate and standardized depiction of the electrode, the quadrant nomenclature of the cochlea introduced by Colby et al. was used [20].

### Image evaluation

All CBCT datasets were assessed subjectively and objectively. Two radiologists with 5 and 10 years of experience in head and neck imaging independently evaluated all CBCT datasets using dedicated PACS viewer (Centricity RIS-i 7.0, GE Healthcare). CBCT datasets were presented in random order with preset window settings chosen according to the subjective preferences of the readers [21, 22]. The readers were blinded to patient identity and imaging acquisition parameters.

The two readers evaluated bone structures of the otic capsule including the osseous spiral lamina, the inner and outer wall of the cochlea, the vestibule and semicircular canals as well as the mastoidal trabeculae. Further, following image criteria were analysed using 5-point Likert scales: Overall image quality (1 = poor image quality, 2 = acceptable image quality, 3 = moderate image quality, 4 = good image quality, 5 = excellent image quality), visibility of the electrode position (1 = the electrode position cannot be determined, 2 = 25%, 3 = 50%, 4 = 75%, 5 = 100% of the electrode position can be determined), visualisation of single electrode contacts (1 = individual contacts cannot be kept apart, 2 = separation of adjacent contacts can be seen at the outer edge, 3 = advanced separation between individual contacts but not complete separation, 4 = some contacts are completely separated from their adjacent contacts, 5 = all contacts are completely separated from their adjacent contacts), osseous spiral lamina (1 = not visible, 2 = vaguely visible, 3 = unambiguously visible, 4 = vaguely visible along the entire length, 5 = unambiguously visible along the entire length), visibility of the inner and outer cochlear wall, the vestibule and semicircular canals, the mastoidal trabeculae (1 = not visible, 2 = vaguely visible but not over the entire length, 3 = vaguely visible over the entire length, 4 = unambiguously visible but not over the entire length, 5 = unambiguously visible over the entire length), and metal and motion artifacts (1 = severe artifacts, image interpretation is impossible, 2 = severe artifacts with strong impairment of image interpretation, 3 = severe artifacts with slight impairment of image interpretation, 4 = slight artifacts without impairment of image interpretation, 5 = no artifacts).

For objective image evaluation, signal-to-noise ratios (SNR) of several anatomic structures were calculated. Circular regions-of-interest of 5 mm diameter were drawn in consistent locations of homogenous bone areas of the temporal bone, of the otic capsule, and the cerebellopontine angle to measure attenuation inmean signal intensities. Image noise was defined as the standard deviation within the background (air). All measurements were performed twice and averaged. Following formula was used for calculating SNR:

SNR = mean signal intensity (Avg) (temporal bone, otic capsule, cerebellopontine angle) / standard deviation of attenuation (SD) (background).

### Radiation dose

Volumetric CT-dose-index (CTDI_VOL_) and dose-area product (DAP) were provided by patient`s protocol. The effective dose (ED) assessments were performed on an anthropomorphic RANDO SK150 phantom (Radiation Analogue Dosimetry System; The Phantom Laboratory, Salem, NY, USA). The measurements were carried out according to a previous study [23] by using a mobile MOSFET device TN-RD- 70-W20 comprising one TN-RD-38 wireless Bluetooth transceiver, four TN-RD-16 reader modules, twenty reinforced high-sensitivity TN-1002RD-H dosimeters and TH-RD-75 M software (Best medical, Ottawa, ON, Canada). Prior to the measurements, the MOSFET dosimeters were calibrated according to previous studies by Koivisto et al. [23, 24]. The ED was calculated from the measured organ doses using the revised guidelines given by the ICRP 103 [25] according to previous study [23].

### Statistical evaluation

Statistical analysis was performed using dedicated statistics software ( R 4.32 and Rstudio 2023.12.13, including the psych libary). Results are presented as mean and standard deviations. The Kolmogorov-Smirnov test was performed to test for normal distribution. Normally distributed variables were analyzed using the unpaired Student`s t-test, for non-normally distributed data the Mann-Whitney-U test was applied. A p-value < 0.05 was considered to be statistically significant, after using Bonferoni correction to adjust for multiple testing. Agreement between the two readers was assessed using the Intraclass Correlation Coefficient (ICC 3 - Single fixed raters). The ICC was interpreted as follows (43): ICC < 0.40, fair; ICC 0.40–0.59, moderate; ICC 0.60–0.74, good; ICC 0.75-1.0, very good agreement.

## Results

We included seventy-five patients (39 females and 36 males; mean age, 55.8 ± 16.5 years; range: 18–85 years). Of those, 32 (42.7%) patients were scanned with the 96-kV protocol and 43 (57.3%) patients with the 120-kV protocol. There were no cases of misinsertion. All 75 CBCT datasets were of diagnostic value without substantial motion artifacts. Cases included CI models from three manufacturers including *Advanced Bionics* (Sonova Holding AG; *n* = 7 [9.3%; 96-kV, *n* = 3; 120-kV, *n* = 4]), *Cochlear* (Cochlear Limited; *n* = 43 [57.3%; 96-kV, *n* = 19; 120-kV, *n* = 24]), and *Med-El* (MED-EL GmbH; *n* = 25 [33.3%; 96-kV, *n* = 10; 120-kV, *n* = 15]) (Fig. 1).

### Subjective image analysis

Table 1 provides the subjective image analysis results. Overall image quality was rated higher in the 120-kV scans compared to the 96-kV (4 [1–5] vs. 3 [2–4], *p* < 0.001). Depiction of nearly all evaluated anatomical structures was rated higher in the 120-kV compared to the 96-kV scans including visualisation of the osseous spiral lamina (3 [1–4] vs. 2 [1–4], *p* < 0.01), visualisation of the outer cochlear wall (4 [1–5] vs. 3 [2–4], *p* = 0.006), differentiation of mastoid bone trabeculae structures (4 [1–5] vs. 3 [2–4], *p* < 0.001) and sharpness of the delineation of the semicircular canals (4 [1–5] vs. 3 [2–4], *p* < 0.001). Depiction of inner cochlear wall rated equal in both settings (3 [1–4] vs. 3 [2–4], *p* = 0.06). Furthermore, 120-kV CBCT provided no better visibility of intracochlear electrode positions with respect to the osseous spiral lamina (4 [1–5] vs. 3.5 [2–5], *p* = 1) as well as for visualization of single electrode contacts per quadrant and in summary over all quadrants (4 [2–5] vs. 3 [1.5-5], *p* = 0.09) compared to 96-kV CBCT. Similar ratings were noted for the presence of metal (3 [1–5] vs. 3 [2–5], *p* = 1) and motion artefacts (4 [1–5] vs. 4 [2–5], *p* = 0.02) with the latter showing a significant difference between the two protocols.

**Table 1:** Subjective image analysis including of cone-beam computed tomography including all cochlear implant models

Rating criteria	96-kV	120-kV	*p*-value
Depiction of …			
osseous spiral lamina	2 [1–4]	3 [1–4]	< 0.001
inner cochlear wall	3 [2–4]	3 [1–4]	< 0.001
outer cochlear wall	3 [2–4]	4 [1–5]	< 0.006
mastoidal trabecular structure	3 [2–4]	4 [1–5]	< 0.001
semicircular canals	3 [2–4]	4 [1–5]	< 0.001
intracochlear electrode position	3.5 [2–5]	4 [1–5]	0.06
single electrode contacts per quadrant	3 [1.5-5]	3.5 [2–5]	0.09
Presence of metal artifacts	3 [2–5]	3 [1–5]	1
Presence of motion artifacts	4 [2–5]	4 [1–5]	0.02
Overall image quality	3 [2–4]	4 [1–5]	< 0.001

Note. Values are median and range (minimum-maximum)

**Fig. 2:**
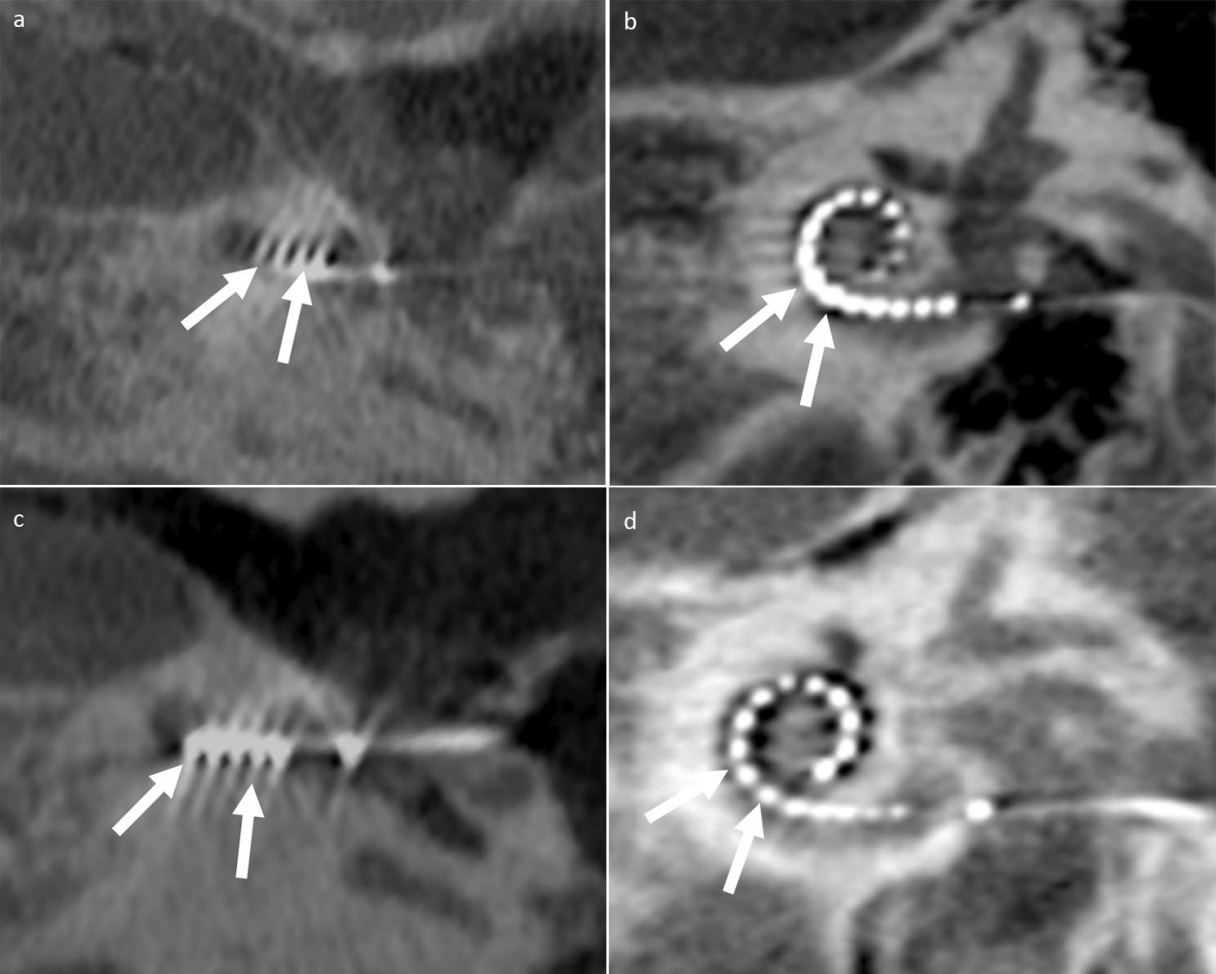
Representative 96-kV (A and C) versus 120-kV (B and D) CBCT axial views (basal turn of the cochlea is shown) and multiplanar reconstructions (Stenvers projection) with inserted Advanced Bionics HiFocus Mid Scala electrodes (Sonova Holding AG). (**A**) and (**B**) showing 96-kV images of a patient with bilateral congenital sensorineural hearing loss after left-sided CI surgery. (**C**) and (**D**) presenting a cochlea view and a Stenvers projection of a patient with unilateral left-sided surditas. 120-kV CBCT provides slightly better overall image quality with better delineation of the electrode contacts and depiction of the adjacent lateral wall of the cochlea compared to 96-kV (white arrows)

**Fig. 3:**
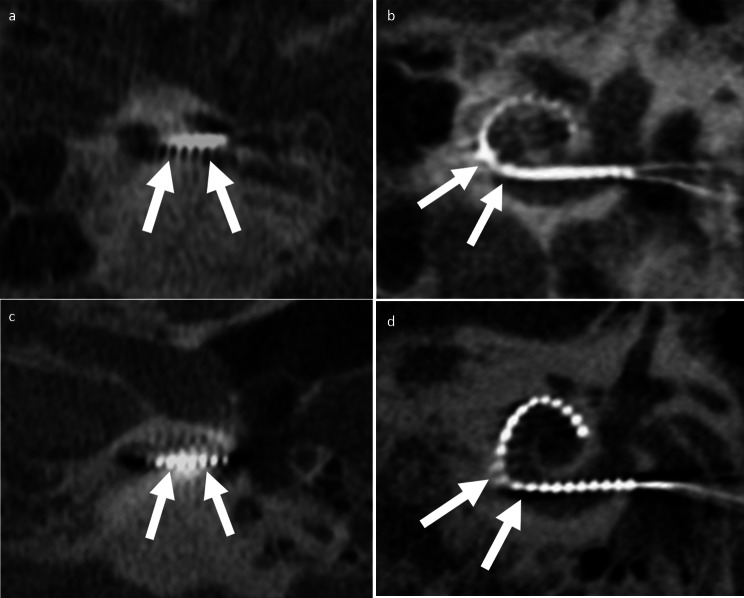
96-kV CBCT axial view of the basal turn of the cochlea (**A**) and a Stenvers projection (**B**) with inserted Slim-Straight CI522 electrode (Cochlear Limited) in patient with bilateral progressive sensorineural hearing loss (right ear shown). (**C**) and (**D**) representing 120-kV CBCT images of a patient with progressive sensorineural hearing loss with inserted Slim-Straight CI522 electrode (Cochlear Limited) on the left side. While 120-kV images had better delineation of electrode contacts (white arrows), all other evaluated image characteristics were rated similar between 96-kV and 120-kV

**Fig. 4:**
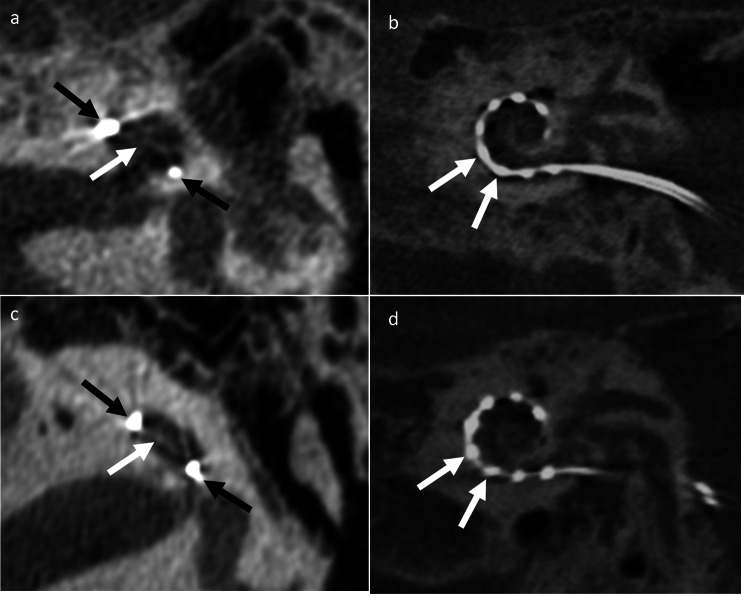
96-kV CBCT (**A** and **B**) of a patient with left-sided progressive sensorineural hearing loss and 120-kV (**C** and **D**) CBCT of a patient with bilateral congenital surditas (right ear shown) in midmodiolar views and Stenvers views multiplanar reconstructions after insertion of MED-EL Flex 28 electrodes (MED-EL GmbH). 120-kV CBCT provides better ratings compared to 96-kV; in particular, the visualization of the osseous spiral lamina (white arrows) and the depiction of the individual electrode contacts per quadrant (black arrows)

Evaluation of CI electrodes only from *Advanced Bionics* (*n* = 7) resulted in an improved overall image quality using 120-kV compared to 96-kV (4 [3–5] vs. 3 [3–4]) with significantly better ratings for the depiction of the outer cochlear wall (4 [3–5] vs. 3 [3–4), the mastoidal trabecular structure (4 [3–5] vs. 3 [3–4]), the semicircular canals (4 [3–5] vs. 3.5 [3–4]), presence of motion artefacts (4 [4–5] vs. 4 [3–5], ) and the intracochlear electrode position (4 [3–5] vs. 4 [2–4]), while osseous spiral lamina, inner cochlear wall and single electrode contacts per quadrant and presence of metal artefacts did not show significant differences (Tables 2**and** Fig. 2). For scans with CI models from *Cochlear*, overall image quality was rated better for 120-kV compared to 96-kV images (3 [1–5] vs. 3 [2–4]). However, the majority of criteria did not show significant differences between 96-kV and 120-kV scans including osseous spiral lamina, intracochlear electrode position, and single electrode contacts per quadrant (Tables 3 and Fig. 3). CBCT scans with CI models from *Med-El* had significant better ratings for overall image quality in 120-kV images compared to 96-kV (4 [3–5] vs. 3 [2–4]) as well as most other evaluated criteria including osseous spiral lamina (3 [2–4] vs. 2 [2–4]) and intracochlear electrode position (4 [3–5] vs. 4 [2–5]), but not for the depiction of single electrode contacts per quadrant (Tables 4and Fig. 4).

**Table 2:** Subjective image analysis of cone-beam computed tomography with cochlear implants from Advanced Bionics (Advanced Bionics LLC).

Rating criteria	96-kV	120-kV
Depiction of …		
osseous spiral lamina	2 [2–3]	3 [2–4]
inner cochlear wall	2.5 [2–3]	3 [2–4]
outer cochlear wall	3 [3–4]	4 [3–5]
mastoidal trabecular structure	3 [3–4]	4 [3–5]
semicircular canals	3.5 [3–4]	4 [3–5]
intracochlear electrode position	4 [2–4]	4 [3–5]
single electrode contacts per quadrant	4 [3–4]	4 [3.5-5]
Presence of metal artifacts	3.5 [2–4]	3.5 [3–5]
Presence of motion artifacts	4 [3–5]	4 [4–5]
Overall image quality	3 [3–4]	4 [3–5]

Note. Values are median and range (minimum-maximum)

**Table 3:** Subjective image analysis of cone-beam computed tomography with cochlear implants from Cochlear (Cochlear Ltd)

Rating criteria	96-kV	120-kV
Depiction of …		
osseous spiral lamina	2 [1–4]	2 [1–4]
inner cochlear wall	3 [2–4]	3 [1–4]
outer cochlear wall	3 [2–4]	4 [1–4]
mastoidal trabecular structure	3 [2–4]	3 [1–5]
semicircular canals	3.5 [2–4]	4 [1–5]
intracochlear electrode position	3 [2–4]	3 [1–5]
single electrode contacts per quadrant	2 [1.5-4]	3 [2–4]
Presence of metal artifacts	3 [2–5]	3 [1–4]
Presence of motion artifacts	4 [2–5]	4 [1–5]
Overall image quality	3 [2–4]	3 [1–5]

Note. Values are median and range (minimum-maximum)

**Table 4:** Subjective image analysis of cone-beam computed tomography with cochlear implant electrodes from Med-El.

Rating criteria	96-kV	120-kV
Depiction of …		
osseous spiral lamina	2 [2–4]	3 [2–4]
inner cochlear wall	3 [2–4]	3 [2–4]
outer cochlear wall	3 [2–4]	4 [3–5]
mastoidal trabecular structure	3 [2–4]	4 [2–5]
semicircular canals	3 [2–4]	4 [3–5]
intracochlear electrode position	4 [2–5]	4 [3–5]
single electrode contacts per quadrant	4 [3–5]	4 [3–5]
Presence of metal artifacts	4 [2–5]	4 [2–5]
Presence of motion artifacts	4 [3–4]	4 [3–5]
Overall image quality	3 [2–4]	4 [3–5]

Note. Values are median and range (minimum-maximum)

Overall interreader agreement was good (ICC = 0.62). There was moderate agreement for delineation of the osseous spiral lamina (ICC = 0.56), the inner (ICC = 0.49) and outer (ICC = 0.52) cochlear walls, the trabecular structures of the otic capsule (ICC = 0.53), the semicircular canals (ICC = 0.44), and the presence of motion artefacts (ICC = 0.58). Interreader agreement was good for visibility of electrode position (ICC = 0.71), presence of metal artefacts (ICC = 0.67), and overall image quality (ICC = 0.72) and very good for visibility of individual electrode contacts per quadrant (ICC = 0.81).

### Objective image quality ratings

SNRs of the temporal bone (23.0 ± 5.7 vs. 13.5 ± 5.7, *p* < 0.001) and the otic capsule (20.9 ± 8.0 vs. 14.0 ± 5.7, *p* = 0.002) were significantly higher in 120-kV compared to 96-kV, while SNR of the cerebellopontine angle did not show significant differences (1.6 ± 1.0 vs. 0.9 ± 0.6, *p* = 0.20) (Table 5**)**.

**Table 5:** Results of objective imaging analysis

Signal-to-noise ratio	96-kV	120-kV	*p*-value
Temporal bone	13.5 ± 5.7	23.0 ± 5.7	< 0.001
Otic capsule	14.0 ± 5.7	20.9 ± 8.0	0.002
Cerebellopontine angle	0.9 ± 0.6	1.6 ± 1.0	0.20

Note. Values are mean and standard deviation

### Radiation dose

For the 96-kV CBCT scans, DAP of 894 mGy*cm and CTDI_VOL_ of 6.7 mGy were recorded, while 120-kV protocol had a DAP of 1585 mGy*cm and CTDI_VOL_ of 14.7 mGy.

The effective doses resulted for 96 kV, 71 mAs: 0.08 mSv and for 120 kV, 71 mAs: 0.15 mSv respectively, *p* < 0.001). The effective dose observed 96 kV was 43% of that acquired for 120 kV.

## Discussion

This retrospective study showed that the depiction of anatomical structures of the inner ear and the evaluation of intracochlear electrode position after CI surgery were improved in the newly available 120-kV CBCT protocol of the temporal bone compared to standard 96-kV CBCT protocol at the expense of a substantially increased radiation exposure.

CBCT is an established imaging modality after CI implantation. Assessment of correct intracochlear location of the electrodes after CI surgery is the main focus of postoperative imaging [18–20]. Accurate delineation of the inner and outer cochlear wall as well as the osseous spiral lamina is essential. A main factor on the visual impact for the assessment of anatomical landmarks of the inner ear depends on their distance to CI electrodes and varies between different CI electrode models and manufacturers [21]. Our study showed that anatomical structures of the cochlear were better visualized using 120-kV CBCT protocol. In scans with CI electrodes from the manufacturers *Advanced Bionics* and *Med-El*, 120-kV CBCT provided better delineation of cochlear structures including the depiction of the osseous spiral lamina as well as depiction of the intracochlear electrode position. In scans with CI electrodes from *Cochlear*, depiction of anatomical structures of the temporal bone improved in 120-kV images compared to 96-kV, while depiction of intracochlear electrode position and single electrodes contacts per quadrant had similar ratings in 96-kV and 120-kV scans. However, 96-kV images are similarly sufficient for postoperative imaging after CI surgery and do not lack any necessary information. There may be future CI models with different, potentially smaller design, that may require imaging with 120-kV, especially models with less inter-contact space. So far, both protocols acquire good images to safely evaluate CI electrode position and rule out insertion trauma.

The occurrence or avoidance of electrode insertion trauma plays an important role to preserve residual hearing and hearing results after CI surgery [22, 26–28]. Therefore, early detection of severe insertion trauma such as electrode penetration into the scala vestibuli or fracture of the osseous spiral lamina has become major task of high-resolution imaging. Wardrop et al. and Ruivo et al. emphasized the close relationship between the electrode position and the occurrence of insertion trauma [29, 30]. In a comparative study of different electrodes brands including *Advanced Bionics, Cochlear* and *Med-El*, Eshraghi et al. demonstrated that even small deviations in the intracochlear penetration depth may lead to insertion trauma of varying degree [31]. While depiction of electrode position in relation to the osseous spiral lamina was significantly improved in 120-kV CBCT for CI electrodes from *Advanced Bionics* and *Med-El*, there was no improvement in 120-kV imaging for CI electrodes from Cochlear. As the density of the contacts of the electrode arrays is higher than density in the inter-contact space, we assume that the missing improvement of electrode depiction in 120-kV CBCT scans of *Cochlear* CI models is due to their design with smaller inter-contact spacing of only 0.4 to 0.9 mm on the CI electrode in comparison to a wider inter-contact spacing on electrodes from *Advanced Bionics* with approximately 1 mm and from *Med-El* with approximately 1.9 to 2.1 mm [32]. Higher tube voltages would lower metal artefacts and may overcome potential diagnostic limitations in CI models with small inter-contact spacing. To further this line of thought, it is worth remembering that the temporal bone is the densest and most stable bone in the human body and is also surrounded by other very dense bones at the base of the skull. Therefore, higher kV-values, that means higher energy photons, are required to avoid attenuation by these dense bones and to achieve better image quality. The signal is amplified relative to the noise. The same applies to the metal in cochlear implants, as beam hardening artefacts are also reduced by higher beam energies [33].

CBCT has been described to allow significant reductions of radiation exposure [34–36]. Theunisse et al. showed that CBCT offers a radiation dose reduction by a factor of 1.6 to 4 compared to CT depending on the CI model with effective doses between 0.04 and 0.10 mSv for CBCT compared to 0.16 mSv for CT [11]. Similar to an increase of tube voltage in CT, the evaluated 120-kV CBCT protocol had a 43% higher radiation exposure compared to the standard 96-kV protocol with exposure values of the 120-kV protocol similar to radiation doses of CT exams of the temporal bone [11]. However, it should be noted that in the present study the adsorbed dose was estimated by conversion between DAP and effective dose based on a dose table applied by the manufacturer and based on a phantom study. Due to the complex dose distribution of CBCT, it is not yet possible to adequately compare common CT dose indices. Alternative dose indices have already been proposed in studies, some of which take into account the geometric aspects of a CBCT scan and represent promising approaches for special conversion formulas between dose-area product and effective dose [37]. Nevertheless, there is substantial increase of radiation exposure is of high impact in those patients, as CI treatment is commonly performed in younger patients who may undergo repeated scans in case of bilateral CI surgery or in case of suspected electrode misplacement, resulting in a higher cumulative radiation exposure. While 120-kV CBCT protocol significantly improved anatomical and CI electrode depiction for CI models from *Advanced Bionics* and *Med-El*, there was no improvement in *Cochlear*’s CI models. Thus, increased radiation exposure does not provide additional information in CBCT imaging after CI surgery using models from *Cochlear*. So far, individualized exam protocols are not typically for CBCT, but should be considered - at least based on the implanted CI model - to find a balance between improved imaging using 120-kV protocol for some CI models and increased risk for radiation-induced malignancies [38–45].

This study has several limitations: First, all patients were examined using the same, new-generation CBCT device. Models of other manufacturers may show variations in design, technical specifications and exam protocols. Therefore, comparison with prior studies and scanners from other manufacturers is limited. This also includes limitations of the scan protocol with a fixed tube current during the scan. Second, subgroup-analysis of image quality between different CI models from the same manufacturer could not be performed due to the limited number of patients. However, models from the same manufacturer have a similar design with comparable effects on image quality, and have therefore been summarized for a manufacturer-based evaluation. Third, subjective image quality was analyzed as part of the quality assessment but did not include detection and evaluation of pathologies. A study including a bigger patient cohort in a prospective design may overcome the mentioned limitations of this study. Furthermore, no children were included in our study. However, children are a big group of patients with need for CI treatment and, thus, require careful evaluation, especially considering the substantially increased radiation exposure of the evaluated 120-kV CBCT protocol.

In summary, our data showed that both, 96-kV and 120-kV CBCT scans provide sufficient diagnostic images of the postoperative temporal bone. There is a substantial benefit of 120-kV CBCT scans assessing the temporal bone regarding better delineation of anatomic structures of the osseous labyrinth and electrode-related aspects after implantation of CI models from *Advanced Bionics* and *Med-El*, despite substantially increased radiation exposure. In comparison, post-surgery 120-kV CBCT of CI models from *Cochlear* did not provide better imaging of anatomical structures and CI electrode. However, the 120-kV CBCT protocol should be chosen carefully with respect to the implanted CI model due to increased radiation exposure, especially in the relatively young CI patient cohort.

## Data Availability

Data and materials are available on request in charge of Dr. med. Ibrahim Yel, Department of Diagnostic and Interventional Radiology, University Hospital Frankfurt, Theodor-Stern-Kai 7, 60590 Frankfurt, Germany and Dr. Hanns Ackermann, Institute of Biostatistics and Mathematical Modeling, University Hospital Frankfurt, Frankfurt, Germany.

## Abbreviations

CI: Cochlear Implant
CBCT: Cone-Beam Computed Tomography
